# Ventricular-arterial uncoupling in heart failure with preserved ejection fraction after myocardial infarction in dogs - invasive versus echocardiographic evaluation

**DOI:** 10.1186/1471-2261-10-32

**Published:** 2010-06-29

**Authors:** Myrielle Mathieu, Bachar El Oumeiri, Karim Touihri, Ielham Hadad, Maryam Mahmoudabady, Philippe Thoma, Thierry Metens, Jozef Bartunek, Guy R Heyndrickx, Serge Brimioulle, Robert Naeije, Kathleen Mc Entee

**Affiliations:** 1 Department of Physiology and Pathophysiology, Faculty of Medicine, ULB, Brussels, Belgium; 2 Cardio-Thoracic Surgery Department, Mont Godinne Hospital, UCL, Yvoir, Belgium; 3 Department of Radiology and Medical Imaging, ULB, Brussels, Belgium; 4 Cardiovascular Center, OLV Hospital, Aalst, Belgium; 5 Faculty of Biomedical engineering, TU Eindhoven, The Netherlands

## Abstract

**Background:**

Heart failure with preserved left ventricular ejection fraction and abnormal diastolic function is commonly observed after recovery from an acute myocardial infarction. The aim of this study was to investigate the physiopathology of heart failure with preserved ejection fraction in a model of healed myocardial infarction in dogs.

**Methods:**

Echocardiography, levels of neurohormones and conductance catheter measurements of left ventricular pressure-volume relationships were obtained in 17 beagle dogs 2 months after a coronary artery ligation, and in 6 controls.

**Results:**

Healed myocardial infarction was associated with preserved echocardiographic left ventricular ejection fraction (0.57 ± 0.01, mean ± SEM) and altered Doppler mitral indices of diastolic function. NT-proBNP was increased, aldosterone was decreased, and norepinephrine was unchanged. Invasive measurements showed a markedly decreased end-systolic elastance (2.1 ± 0.2 vs 6.1 ± 0.8, mmHg/ml, p < 0.001) and end-systolic elastance to effective arterial elastance ratio (0.6 ± 0.1 vs 1.4 ± 0.2, p < 0.001), with altered active relaxation (dP/dtmin -1992 ± 71 vs -2821 ± 305, mmHg/s, p < 0.01) but preserved left ventricular capacitance (70 ± 6 vs 61 ± 3, ml at 20 mmHg, p = NS) and stiffness constant. Among echocardiographic variables, the wall motion score index was the most reliable indicator of cardiac contractility while E', E/A and E'/A' were correlated to dP/dtmin.

**Conclusions:**

In the canine model of healed myocardial infarction induced by coronary ligation, heart failure is essentially characterized by an altered contractility with left ventricular-arterial uncoupling despite vascular compensation rather than by abnormal diastolic function

## Background

Heart failure with preserved left ventricular (LV) systolic function as assessed by echocardiographic measurement of an ejection fraction (EF) higher than 50%, together with altered indices of diastolic function [1], is observed after acute myocardial infarction in 30 to 50% of heart failure patients [2,3]. In heart failure with preserved LV systolic function, increased LV filling pressure is generally attributed to abnormalities in active relaxation and passive stiffness caused by the combination of ischemia-induced alteration in relaxation, myocardial hypertrophy and fibrosis and leading to diastolic dysfunction [4,5]. However, unlike the observations in hypertensive heart disease, there is limited direct evidence of intrinsic alteration of LV diastolic function i.e. invasive demonstration of increased diastolic stiffness in patients who recovered from an acute myocardial infarction. In experimental myocardial infarction in rats, the LV diastolic pressure-volume curve was initially shifted to the left, to be later-on displaced to lower pressures and higher volumes, suggesting rather an increased LV diastolic compliance [6]. In dogs with heart failure and preserved ejection fraction induced by coronary microembolization, increased LV end-diastolic pressure was explained by neurohumoral activation and volume expansion rather than by a change in LV end-diastolic pressure-volume relationship [7]. The authors concluded that heart failure with preserved EF does not a priori equate with diastolic heart failure.

In order to explain the pathophysiological mechanism (diastolic versus systolic heart failure) of elevated diastolic pressures, we investigated systolic and diastolic function by invasive studies of LV pressure-volume relationships in dogs, two months after recovery from myocardial infarction induced by a coronary ligation. We also recorded indices of systolic and diastolic function by Doppler echocardiography and tissue Doppler imaging, and measured circulating neurohormones N-terminal B-type natriuretic propeptide (NT-proBNP), aldosterone and norepinephrine, to investigate their potential value in predicting systolic and diastolic alterations in LV function.

## Methods

### Animal model

The protocol was approved by the Animal Care and Use Committee of the ULB, and conformed to the "Guide for the Care and Use of Laboratory Animals published by The US National Institutes of Health (NIH Publication No. 85-23, revised 1996).Twenty-three beagles (CEDS, Mézilles, France), weighing 13.7 ± 0.4 kg and aged 12 ± 2 months, were included in the study. Myocardial infarction was induced in 17 dogs, 6 were used as controls. After premedication with midazolam (0.1 mg/kg IV) and sufentanyl (0.1 μg/kg IV), anesthesia was induced with propofol (3 - 4 mg/kg IV) and maintained with isoflurane (1.5 - 2%). Animals were ventilated with a FiO_2 _of 1, a respiratory rate of 12 cycles/min and a tidal volume of 12 - 15 ml/kg. Lactate-Ringer was infused at 10 ml/kg/hour and sufentanyl at 1 μg/kg/hour. During anesthesia, care was taken to maintain body temperature above 36°C. A left thoracotomy was performed, the pericardium opened, and the left circumflex artery and/or its marginales dissected and permanently ligated with special care to obtain the same infarction extent despite individual anatomical variations. This procedure was realized under constant ECG monitoring, and ventricular arrhythmias were treated with lidocaïne (2 to 4 mg/kg IV over a period of 2 minutes followed by an infusion of 75 μg/kg/min). After surgery, buprenorphine (10 μg/kg IM, 3 times a day) and cefalexin (20 mg/kg SC, 2 times a day) were administered during 48 hours.

### Study design

A clinical examination including cardiac auscultation and a Doppler-echocardiography was performed before inclusion of the dogs in the study. Blood for electrolytes and neurohormones measurements and echocardiography were performed in the conscious state 1 week before coronary ligation (baseline) and repeated 2 months after recovery from myocardial infarction. A cardiac magnetic resonance imaging (MRI) and a left heart catheterization for the measurement of pressure-volume loops were performed 2 months after myocardial infarction. We also realized pressure-volume loops in 6 weight- and age-matched control dogs.

### Magnetic resonance imaging

Infarct size was assessed by using the 1.5 Tesla clinical MRI scanner (Achieva 1.5 T; Philips Best, The Netherlands) with a 2-element phased array coil placed over the chest (Sense Flex-M Coil; Philips, Brussels, Belgium). Gadodiamide (0.2 mmol/kg) was manually injected intravenously in 3 seconds. Following a delay of 10 to 15 minutes, contrast-enhanced images were acquired and raw images processed by manual outlining of the endocardial, epicardial and infarct borders. Total infarct volume was calculated as the summation of the contrast-enhanced volumes from the entire left ventricular myocardial volume. Relative infarction size was determined as the ratio of infarct volume to LV wall volume times 100.

### Echocardiography

In order to obtain high quality Doppler-echocardiographic images in an unstressed state, the dogs had been acclimatized to the laboratory and trained to lay in lateral recumbency on a table. Doppler echocardiography (Vivid 5, GE, Brussels, Belgium) was carried out under continuous ECG monitoring with a 5 MHz electronic probe, the dog, in conscious state, lying in lateral recumbency, scanning through the dependent chest wall through an contrived hole in the table. A right parasternal window was used to record bidimensional and M-mode images in the long and short axes. A left parasternal window was used to record the bidimensional apical four chambers view, pulsed-wave Doppler of mitral flow and tissue Doppler of mitral septal annulus movement. The LV length and endocardial borders were traced at end-diastole and end-systole, using the bidimensional right long axis view which best shows the LV cavity, in order to calculate end-systolic and end-diastolic LV volumes (LVEDV_echo_, LVESV_echo_) and ejection fraction (LVEF_*echo*_) by the single plane Simpson's rule. Fractional shortening (FS) was calculated from M-mode derived linear dimensions and LV mass was derived from the truncated ellipsoid model. Relative wall thickness (RWT) was calculated in diastole as (posterior wall thickness + interventricular septum thickness)/(LV internal diameter). Early diastolic myocardial velocity (E'), late (atrial contraction) diastolic myocardial velocity (A'), and systolic myocardial velocity (S') of the septal annulus movement were measured and the E'/A' ratio calculated. Additional Doppler assessment included the peak velocities of the mitral flow E and A waves (E and A), mitral flow deceleration time (DT) and the derived E/A and E/E'ratios. All images were recorded and analyzed off line according to specific guidelines [8,9]. To evaluate regional systolic function, the left ventricle, observed from its apical, mid and basal right short axis views, was divided according to a 16-segment model [9]. For each segment, wall motion was scored as 1 (normal or hyperkinetic segment), 2 (mildly hypokinetic segment), 3 (severely hypokinetic segment), 4 (akinetic segment) and 5(dyskinetic segment) and the wall motion score index (WMSI) was derived as the ratio between the sum of all scores and the number of observed segments.

### Pressure-volume loops

Anesthesia was induced by intravenous injection of midazolam 1 mg/kg (Dormicum, Roche, Brussels, Belgium) and sufentanyl 3 μg/kg (Sufenta forte, Janssen-Cilag, Berchem, Belgium)and maintained by continuous infusion (midazolam 0.750-1.125 mg/kg/hour + sufentanyl 2.5-3.75 μg/kg/hour). The dogs were mechanically ventilated (FiO_2_: 0.4) and care was taken to maintain end-expiratory CO_2 _between 30 and 35 mmHg and body temperature above 36°C. Under fluoroscopic control, a 6F pigtail combined conductance-pressure catheter (Millar Instruments, Inc, Houston, Texas) was advanced through the carotid into the LV apex and connected to a Leycom Sigma-5 signal processor (Cardiodynamics, Leiden, The Netherlands) for instantaneous LV volume signal and to a Millar pressure monitor (Model TC-510, Millar Instruments Inc, Houston, USA) for instantaneous LV pressure signal. A 5F Swan-Ganz thermodilution catheter (93-132-5F, Baxter, Irvine, USA) was advanced through the jugular vein and positioned in a main branch of the pulmonary artery for cardiac output (CO) measurements. A 7F transluminal valvuloplasty catheter (Tyshak II, Numed, Medical EuropeBV, The Netherlands, balloon diameter: 12 mm, balloon length: 3 cm) was advanced through the jugular vein and positioned in the posterior vena cava just below the junction with the right atrium for transient preload reductions. Blood conductivity was determined using the Rho cuvette. Parallel conductance was measured in duplicate by the hypertonic saline method (7.5 ml NaCl 10%) [10]. Cardiac output was measured in triplicate by the thermodilution technique (5 ml ice-cold dextrose 5%) to determine the α calibration factor of conductance volumetry. Electrocardiographic and hemodynamic signals were digitized at 1000 Hz and 500 Hz respectively. Real time signal processing was obtained on dedicated software (IOX; Emka Technologies SA, Paris, France). All measurements were performed during brief end-expiratory pauses. At least 10 pressure-volume loops were analyzed to assess steady-state hemodynamic signals. End-systolic and end-diastolic pressure-volume relationships (ESPVR and EDPVR) were obtained in triplicate during transient vena cava occlusion. Linear regression analyses of the end-systolic pressure-volume coordinates during transient preload reduction determined the slope (Ees) of the ESPVR. The LV afterload was quantified using effective arterial elastance (Ea) that is the ratio between LV end-systolic pressure (LVESP) and stroke volume (SV). To evaluate ventricular-arterial coupling, the Ees/Ea ratio was calculated [11]. Active relaxation was quantified by dP/dtmin and the time constant of pressure fall (Tau), determined by fitting a mono-exponential curve to the isovolumic period of the ventricular pressure curve. The EDPVR was determined by applying non linear regression analyses (LVEDP = a e^βLVEDV^) to the end-diastolic pressure-volume points. The chamber stiffness constant (β) and capacitances (LV volumes) at pressures of 15 and 20 mmHg were derived from the EDPVR of each dog [12].

### Neurohormones and electrolytes

Commercially available radioimmunoassay kits were used to assess NT-proBNP (VETSIGNCanine CardioSCREEN Nt-proBNP Test VC4010, Guildhay Limited, Guildford, England), aldosterone (Coat-A-Count Aldosterone TKAL1, Siemens Medical Solutions Diagnostics, Brussels, Belgium) and norepinephrine (Noradrenalin Research EIA KAPL10-5200, Biosource, Nivelles, Belgium). Serum sodium, potassium and osmolality levels were measured (Laboratory of Medical Biology Baudouin, Enghien, Belgium).

### Statistical analysis

All results are expressed as mean ± SEM and statistical significance is determined at p < 0.05. Distribution normality was tested with a Kolmogorov-Smirnov test. Normally distributed data were tested by paired or unpaired Student's t-tests. Other data were analyzed by a Mann and Whithney test. Forward stepwise regressions were used to investigate the correlations between echocardiographic variables, neurohormones and invasive measurements [13].

## Results

### Magnetic resonance imaging

The MRI performed 2 months after myocardial infarction revealed that we were in presence of a moderate latero-posterior infarct with a myocardial infarction size of 13 ± 0.74% of the LV wall.

### Echocardiography (Table 1)

**Table 1:** Echocardiographic data

	Baseline (n = 17)	Post MI (n = 17)	p value
***Left ventricular remodeling***			
LVEDV, ml	29 ± 1	36 ± 2	< 0.001
LV mass, g	74 ± 3	95 ± 5	< 0.001
RWT	0.46 ± 0.01	0.36 ± 0.02	< 0.001
***Left ventricular systolic function***			
LVEF, %	0.70 ± 0.01	0.57 ± 0.01	< 0.001
LVESV, ml	8.8 ± 0.6	15.5 ± 1.0	< 0.001
WMSI	1 ± 0.00	1.81 ± 0.06	< 0.001
S', m/s	0.109 ± 0.005	0.079 ± 0.003	< 0.001
FS, %	37 ± 1	33 ± 1	0.014
***Mitral inflow***			
E, m/s	0.83 ± 0.03	0.77 ± 0.02	0.069
A, m/s	0.56 ± 0.02	0.64 ± 0.02	0.036
DT, s	0.079 ± 0.003	0.080 ± 0.001	0.838
E/A	1.51 ± 0.05	1.22 ± 0.05	< 0.001
***Mitral annulus displacement***			
E', m/s	0.099 ± 0.008	0.074 ± 0.003	0.015
A', m/s	0.072 ± 0.005	0.074 ± 0.003	0.668
E'/A'	1.41 ± 0.12	1.03 ± 0.07	0.01
E/E'	8.4 ± 0.5	10.4 ± 0.6	0.044

Values are expressed as mean ± SEM. LV: left ventricle; EDV: end-diastolic volume; RWT: relative wall thickness; EF: ejection fraction; ESV: end-systolic volume; WMSI: wall motion score index; S': systolic myocardial velocity of septal mitral annulus; FS: fractional shortening; E and A: maximal velocities of the mitral E and A waves; DT: deceleration time of early mitral flow; E': early diastolic myocardial velocity of septal mitral annulus; A': late diastolic myocardial velocity of septal mitral annulus.

*Left ventricular remodeling - *As compared to baseline, the infarcted dogs showed increased LVEDVecho and LVmass and a decreased RWT.

*Left ventricular systolic function *- The LVEF_echo _was decreased but its mean value remained above 50%. The LVEF_echo _stayed higher than 50% in all infarcted dogs except 3 and higher than 45% in all dogs 2 months after myocardial infarction. The FS was mildly reduced but remained above 25%, mitral S' was reduced, while LVESV_echo _and WMSI were increased.

*Left ventricular diastolic function *- E', E/A and E'/A' were decreased, E, A' and DT stayed unchanged and A and E/E' were increased.

### Pressure-volume loops (Table 2)

**Table 2:** PV loops data

	Control (n = 6)	Post MI (n = 17)	P value
***Global cardiac function***			
HR, beats/min	81 ± 4	79 ± 3	0.319
CO, l/min	2.7 ± 0.3	2.7 ± 0.2	0.941
MAP, mmHg	111 ± 5	89 ± 2	< 0.001
LVEDP, mmHg	14 ± 1	23 ± 1	< 0.001
***Preload and afterload***			
LVEDV, ml	40 ± 5	65 ± 3	0.002
Ea, mmHg/ml	4.4 ± 0.5	3.4 ± 0.2	0.05
***Systolic left ventricular function***			
LVEF, %	64 ± 2	50 ± 2	< 0.001
LVESV, ml	19 ± 2	36 ± 2	< 0.001
dP/dt max, mmHg/s	3677 ± 368	2568 ± 105	< 0.001
Ees, mmHg/ml	6.1 ± 0.8	2.1 ± 0.2	< 0.001
V0, ml	-5 ± 2	-23 ± 9	0.113
***Ventricular-arterial coupling***			
Ees/Ea	1.4 ± 0.2	0.6 ± 0.1	< 0.001
***Diastolic left ventricular function***			
*Active relaxation*			
Tau, ms	29 ± 5	36 ± 2	0.057
dP/dtmin, mmHg/s	-2821 ± 305	-1992 ± 72	0.007
*Passive diastolic properties*			
β, 1/ml	0.053 ± 0.006	0.075 ± 0.011	0.191
Capacitance (15 mmHg), ml	55 ± 3	66 ± 5	0.177
Capacitance (20 mmHg), ml	61 ± 3	70 ± 6	0.249

Values are expressed as mean ± SEM. HR: heart rate; CO: cardiac output; MAP: mean arterial pressure; LV: left ventricular; ESV: end-systolic volume, EF: ejection fraction; Ees: end-systolic elastance; V0: volume intercept; Tau: time constant of relaxation; β, the chamber stiffness constant, is derived from the EDPVR equation (LVEDP = α e^βLVEDV^); EDV: end-diastolic volume; EDP: end-diastolic pressure; Ea: arterial elastance.

**Figure 1:**
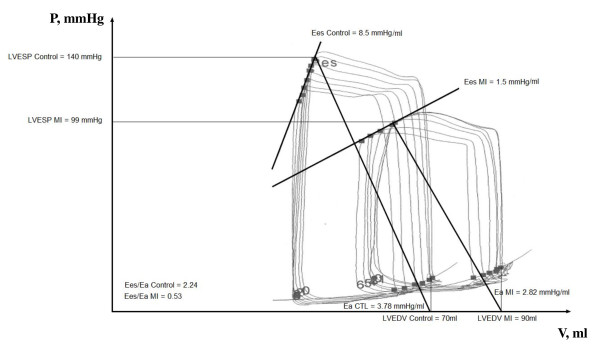
**PV loops under preload reduction**. Representative left ventricular (LV) pressure-volume (PV) loops during vena cava occlusion in a control dog and in a dog 2 months after recovery from a myocardial infarction. Recovery from myocardial infarction was associated with a decreased end-systolic elastance (decreased contractility, altered systolic function) but unchanged/slightly decreased diastolic elastance (unchanged diastolic function). Ees: end-systolic pressure volume relationship; Ea: effective arterial elastance; MI:myocardial infarction; ESP: end-systolic pressure; EDV: end-diastolic volume.

*Left ventricular global and systolic function *- In infarcted dogs as compared to controls, LVEDP, LVEDV, and LVESV were increased, while LVEF, dP/dtmax, mean arterial pressure, Ees and Ea were decreased, whereas, heart rate and CO stayed unchanged. It is of interest that Ees decreased proportionally more than Ea, resulting in a decrease in Ees/Ea. Representative pressure-volume loops during transient vena cava occlusion, Ees and Ea in a control dog and in a dog 2 months after myocardial infarction are shown in figure 1.

*Left ventricular diastolic function *- The infarcted dogs had a reduced dP/dtmin and Tau tended to increase (p = 0.057). Capacitances at 15 mmHg and 20 mmHg and the chamber stiffness constant β remained unchanged in infarcted dogs compared to controls.

### Neurohormonal activation and electrolytic balance (Table 3)

**Table 3:** Neurohormones and electrolytes

	Baseline (n = 17)	Post MI (n = 17)	P value
NT-proBNP, pmol/l	218 ± 36	547 ± 78	< 0.01
Aldosterone, pg/ml	91 ± 19	37 ± 12	< 0.01
Norepinephrine, pg/ml	63 ± 3.0	62 ± 3.0	NS
Na^+^, mmol/l	148 ± 1.0	148 ± 1	NS
K^+^, mmol/l	4.7 ± 0.1	4.5 ± 0.2	NS
Osmolality, mOsmol/kg	293 ± 1.0	292 ± 1	NS

Values are expressed as mean ± SEM. NT-proBNP: N-terminal B-type natriuretic propeptide.

Compared to baseline, NT-proBNP was increased, aldosterone was decreased, and norepinephrine was unchanged after recovery from myocardial infarction. Serum electrolytes and osmolality stayed unchanged after myocardial infarction.

### Non invasive prediction of systolic function

**Figure 2:**
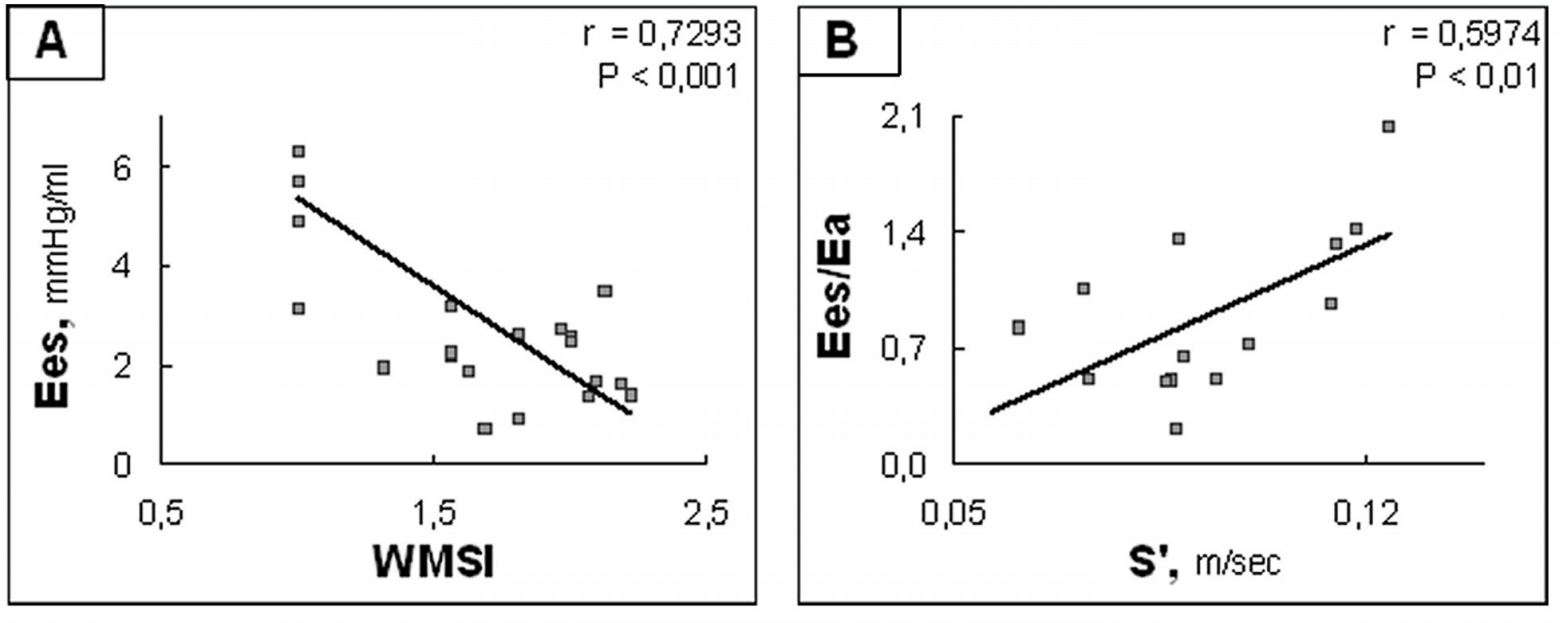
**Correlations between hemodynamic and echocardiographic data: systolic function**. A: Scatterplot of Ees as a function of WMSI. B: Scatterplot of Ees/Ea as a function of S'. Ees: left ventricular end-systolic elastance; WMSI: wall motion score index; Ea: arterial elastance; S': Tissue Doppler mitral annulus S wave.

The echocardiographic indices of systolic function WMSI, LVEF, S' and LVESV were correlated to Ees (r = 0.73, p < 0.001 for WMSI, r = 0.71, p < 0.001 for LVEF, r = 0.71, p < 0.01 for S' and r = 0.59, p < 0.01 for LVESV) and to Ees/Ea (r = 0.61, p < 0.01 for WMSI, r = 0.60, p < 0.01 for S' and r = 0.58, p < 0.01 for LVEF) while FS was not correlated to Ees (r = 0.25, p = NS) and FS and LVESV were not correlated to Ees/Ea (r = 0.33, p = NS for FS and r = 0.29, p = NS for LVESV). Forward stepwise regression analyses showed that the best predictors of Ees and Ees/Ea were respectively WMSI (r = 0.74, p < 0.001) and S' (r = 0.60, p = 0.024) (Figure 2). NT-proBNP was correlated to Ees (r = 0.46, p < 0.05) but not to Ees/Ea (r = 0.34, p = NS).

### Non invasive prediction of diastolic function

**Figure 3:**
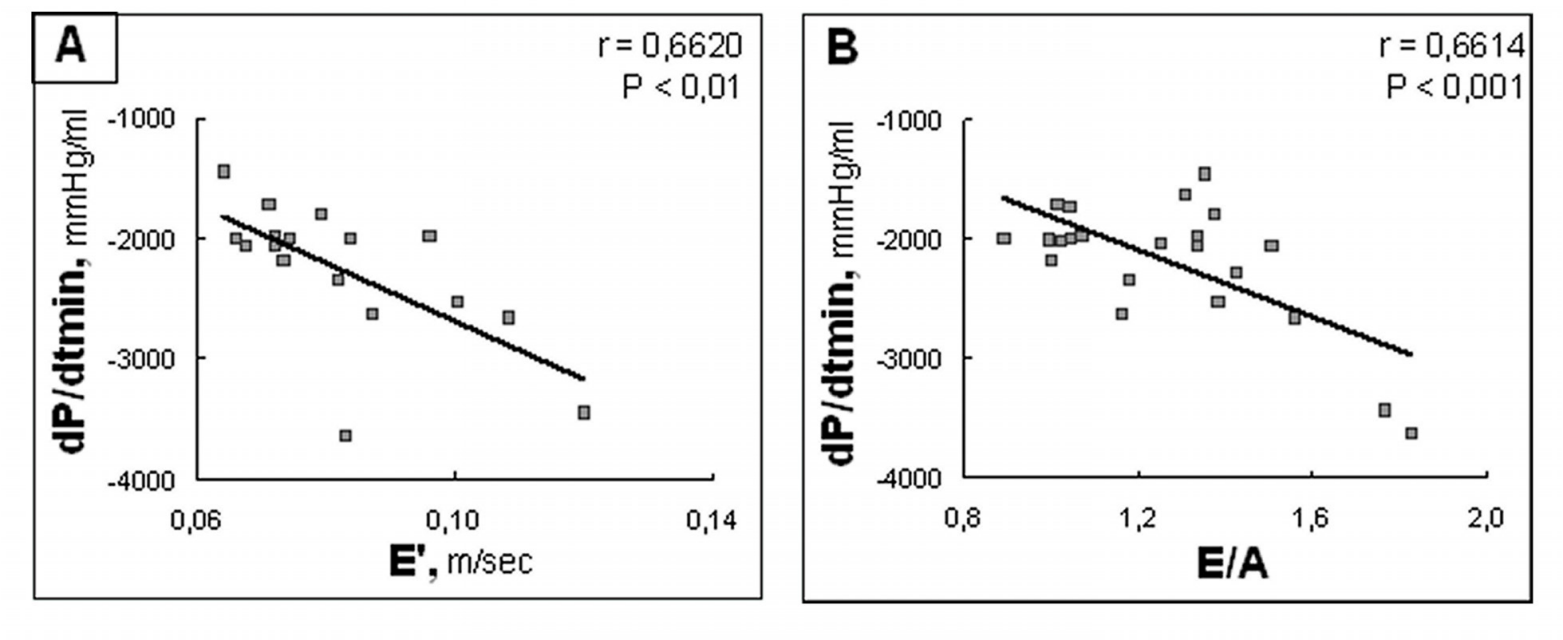
**Correlations between hemodynamic and echocardiographic data: active relaxation**. A: Scatterplot of dP/dtmin as a function of tissue Doppler mitral annulus velocity E' wave. B: Scatterplot of dP/dtmin as a function of mitral Doppler flow ratio of E and A waves.

There was no correlation between E, A, E/A, E', A', E'/A' or E/E' and LVEDP, capacitances at 15 mmHg and 20 mmHg. However, E/A, E' and E'/A' were correlated to dP/dtmin (r = 0.66, p < 0.001 for E/A, r = 0.66, p < 0.01 for E' and r = 0.52, p < 0.05 for E'/A') but not to Tau. Forward stepwise regression analysis showed that E/A and E' were the best predictors of active relaxation determined by dP/dtmin (partial r = 0.69, multiple r = 0.78, p <0.001) (Figure 3).

## Discussion

In the present study, the dogs with healed myocardial infarction presented with a stage B heart failure, defined as a structural disease strongly associated with the development of heart failure but without signs or symptoms at rest [14]. The echocardiographic profile was suggestive of altered diastolic function, with abnormal transmitral flow pattern and mitral annulus velocities, while LVEF_echo _stayed above 50%. Therefore the heart failure qualified for a diagnosis of "heart failure with preserved ejection fraction".

A decreased end-systolic elastance (Ees) and/or a rightward shift of the end-systolic pressure-volume relationship (ESPVR) are characteristics of decreased inotropism. Acute regional left ventricular ischemia has been shown to induce a rightward shift of the ESPVR without any change in Ees [15,16] or a reduced Ees with or without rightward shift of the ESPVR [17]. In our study, we observed a reduced Ees compared to control dogs while no significant change in the volume axis intercept (V0) was obtained. Chronic regional ischemia induces cardiac remodeling in the infarct and border zones but also in the remote zone [18] leading to a stage of dilated ischemic cardiomyopathy. Our results are in accordance with those of Ahmet et al (2004) who showed a reduction of Ees in a rodent model of dilated ischemic cardiomyopathy, 8 weeks after coronary ligation, [19].

The adequacy of systolic function adaptation to afterload can be evaluated by a ratio of end-systolic ventricular and arterial elastances, Ees/Ea [11]. Extensive experimental work and modeling has established that the ratio of elastances allowing for optimal ventricular-arterial coupling, or flow output at a minimal energy cost, is approximately of 2 [20]. Control values for Ees/Ea in the present experiments were lower, around 1.4, but in keeping with reported values in chronically instrumented conscious dogs [7,21] and higher than values reported in conscious healthy humans [22,23]. This ratio, used for index of the ventricular-arterial coupling, takes into account the capability of a ventricle to adapt its intrinsic contractile state to afterload. In the present study, heart failure with "preserved" LVEF was associated with a three-fold decrease in Ees, but the decrease in Ees/Ea, though important, was proportionally less important because of concomitantly decreased Ea. These changes underscore the severity of systolic failure and ventricular-arterial uncoupling occurring after the healing of a limited size myocardial infarction. However, vascular compensation (decreased afterload) allowed EF to be rather well maintained and led to an underestimation of systolic dysfunction by echocardiographic measurements.

We wondered if general anesthesia applied during the invasive measurements could have accounted for an overestimation of systolic failure and ventricular-arterial uncoupling in the present experiments. Both midazolam and sufentanyl have cardiodepressant and hypotensive effects [24,25]. Both could decrease Ees and Ea, although their net effect on ventricular-arterial coupling might be difficult to predict. Compared to measurements reported in conscious chronically instrumented normal dogs before and after coronary microembolization [7], Ea in the present experiment was more decreased, accounting for higher Ees/Ea suggesting that the anesthetic protocol used in the present experiments could have led to relatively improved load dependent indices of systolic function and ventricular-arterial coupling compared to the conscious state. Moreover, the decrease in LVEF observed after myocardial infarction was actually of the same magnitude (-13 to 14%) as assessed invasively as well as non invasively. Thus, even though we cannot exclude specific effects on diastolic function, it appears reasonable to assume that general anesthesia in the present experiments did not affect the results.

Measurements of Ees and Ees/Ea and associated diastolic function changes in heart failure with preserved LVEF have been reported variably according to underlying physiopathology. In patients with various non ischemic cardiac diseases, Ees and Ea tend to be preserved, but diastolic stiffening is a constant finding [4,5,22]. Systolic hypertension has been reported to be associated with both increased Ees and Ea, but with diastolic abnormalities including an upward-shifted diastolic pressure-volume curve [23]. This is in opposition to what is found in the present study that relates to ischemic heart disease. Heart failure after an acute myocardial infarction is initially systolic [23] but often evolves to recovery of LVEF but persistently abnormal indices of diastolic function [2,3]. Invasive measurements of pressure-volume loops in patients with ischemic cardiomyopathy and heart failure with or without depressed LVEF are scarce. A study on patients with an old myocardial infarction and a depressed LVEF (to an average of 37%) showed a marked decrease in the Ees/Ea ratio to 0.7 mainly because of an increased Ea [26]. Nifedipine in these patients increased Ees/Ea ratio to 1 by a decrease in Ea. We are not aware of previous reports on Ees/Ea in patients with heart failure and preserved LVEF after healing from a myocardial infarction.

While in our experiments Ees was decreased as expected, there was also a decrease in Ea suggesting an adaptative decrease in arterial stiffness, albeit insufficient to prevent uncoupling. This decrease in Ea, also observed in heart failure after coronary microembolism in dogs [7] could be related to secretion of natriuretic peptides. These have vasorelaxant and natriuretic effects and inhibit the renin-angiotensin-aldosterone and sympathetic systems [27]. In the present study, increased NT-pro-BNP and decreased aldosterone in the face of unchanged norepinephrine and normal serum electrolytes are in keeping with this notion of adaptative vasorelaxant neurohumoral activation. Vasoconstricting neurohumoral activation with predominant sympathetic nervous system activation is more generally reported in heart failure, probably in relation to more advanced and symptomatic disease [28]. But low aldosterone levels associated with activation of ANP has been described in dogs with naturally acquired mitral valve regurgitation at the time of decompensation [29]. An alternative mechanical explanation of the decrease in Ea would be in the non-linearity of elastance curves, allowing for coupling to operate at lower pressure-related improved compliance [30]. Whatever the mechanism, a decrease in Ea helped to a relatively maintained LVEF in the dogs with healed myocardial infarction.

In the present study, most of the echocardiographic indices of systolic function predicted Ees or Ees/Ea, but the most performant of them appeared to be WMSI for Ees and mitral septal annulus S' wave for Ees/Ea. The WMSI reflects healed infarction-related wall motion asynchrony, and its sensitivity in the alteration of systolic function in postmyocardial infarction [31]. It has been shown to be more sensitive in predicting cardiac events after a myocardial infarction than LVEF or exercising testing [32]. Tissue Doppler mitral S' wave is a reflection of atrioventricular plane longitudinal displacement velocity, thought to be more sensitive to systolic function changes than LVEF [33], and as such more frequently abnormal in heart failure with preserved LVEF [34]. In the present study, active relaxation in dogs with healed myocardial infarction was altered as demonstrated by a decreased dP/dtmin and a borderline significant increase in Tau. Slowing of active relaxation expectedly decreased E', E/A and E'/A', echocardiographic indices of diastolic function. The balanced influences of delayed relaxation and increased left atrio-ventricular pressure gradient on DT and E explain that these variables remained stable. It is of interest that, in the present study, without alteration of LV compliance, E', E/A and E'/A' were correlated to dP/dtmin and not to the chamber stiffness constant. This underscores the fact that abnormalities in the mitral filling pattern and mitral annulus movement are not sufficient to diagnose an intrinsic alteration of LV compliance at rest [35]. These results are in contrast with a previous study in patients, with non ischemic heart failure and preserved LVEF, presenting an invasively identified diastolic dysfunction [36]. In this study, in which 79% of patients had arterial hypertension, the authors showed that mitral filling pattern and mitral annulus diastolic velocities were correlated to altered LV compliance, and identified E/E' as the best predictor of increased LV stiffness. Our study differs in etiology and physiopathology of heart failure with preserved LVEF.

## Conclusion

In conclusion, heart failure with preserved LVEF observed in dogs 2 months after an acute myocardial infarction relates to decreased contractility leading to ventricular-arterial uncoupling despite vascular compensation while LV compliance at rest is preserved. In these circumstances the WMSI is the most performant echocardiographic index to predict cardiac contractility while E/A and E'/A' are correlated to LV active relaxation but not to LV compliance.

## Experimental limitation

The pericardium was opened during thoracotomy to ligate the circumflex artery and left opened to avoid any increase in intrapericardial pressure due to postoperative bleeding or exsudation. PV loops mainly depend on ventricular preload, afterload, contractility, relaxation and compliance but may be affected by intrapericardial pressure. In normal hearts, the pericardium is minimally stressed and its effect on LV pressure is small. In acute cardiac dilation, the stress is increased and intrapericardial pressure may substantially contribute to LV diastolic pressure [37]. Over time, the pericardium expands and its effect on PV loops disappears. Lewinter and Pavelec (1982) showed that removing the pericardium after 50 days of volume overload-induced dilation had no effect on LV end-diastolic volume or pressure [38]. We therefore estimated that, in our present study, opening the pericardium would no longer affect PV loops at time of measurements, i.e. 2 months after causing the infarction.

## Competing interests

The authors declare that they have no competing interests.

## Authors' contributions

MM carried out the coronary ligations, the pressure-volume loops (recording and analysis) and the magnetic resonance imaging (analysis), performed the statistical analysis and drafted the manuscript. BE carried out the coronary ligations. KT anesthetized the dogs for the coronary ligations, the pressure-volume loops and the magnetic resonance imaging. IH and MM participated in the serum BNP dosages. PT and TM carried out the resonance imaging recording and analysis. JB and GH participated in the coordination of the study and helped to draft the manuscript. SB and RN participated in the analysis of the pressure-volume loops results and helped to draft the manuscript. KME conceived the study, participated in its design, coordinated the study, carried out the echocardiography, and helped to draft the manuscript. All authors read and approved the final manuscript.

## Pre-publication history

The pre-publication history for this paper can be accessed here:

http://www.biomedcentral.com/1471-2261/10/32/prepub
